# Inhibition of the insulin-like growth factor-1 receptor potentiates acute effects of castration in a rat model for prostate cancer growth in bone

**DOI:** 10.1007/s10585-017-9848-8

**Published:** 2017-04-26

**Authors:** Annika Nordstrand, Sofia Halin Bergström, Elin Thysell, Erik Bovinder-Ylitalo, Ulf H. Lerner, Anders Widmark, Anders Bergh, Pernilla Wikström

**Affiliations:** 1 Department of Radiation Sciences, Oncology, Umeå University, Umeå, Sweden; 2 Department of Medical Biosciences, Pathology, Umeå University, Umeå, Sweden; 3 Department of Molecular Periodontology, Umeå University, Umeå, Sweden; 4 Centre for Bone and Arthritis Research, Department of Internal Medicine and Clinical Nutrition at Institute for Medicine, Sahlgrenska Academy at University of Gothenburg, Gothenburg, Sweden

**Keywords:** Bone metastasis, IGF-1R, Apoptosis, Proliferation, Immune response, RUNX2, TRAP

## Abstract

Prostate cancer (PCa) patients with bone metastases are primarily treated with androgen deprivation therapy (ADT). Less pronounced ADT effects are seen in metastases than in primary tumors. To test if acute effects of ADT was enhanced by concurrent inhibition of pro-survival insulin-like growth factor 1 (IGF-1), rats were inoculated with Dunning R3327-G tumor cells into the tibial bone marrow cavity and established tumors were treated with castration in combination with IGF-1 receptor (IGF-1R) inhibitor NVP-AEW541, or by each treatment alone. Dunning R3327-G cells were stimulated by androgens and IGF-1 in vitro. In rat tibia, Dunning R3327-G cells induced bone remodeling, identified through increased immunoreactivity of osteoblast and osteoclast markers. Tumor cells occasionally grew outside the tibia, and proliferation and apoptotic rates a few days after treatment were evaluated by scoring BrdU- and caspase-3-positive tumor cells inside and outside the bone marrow cavity, separately. Apoptosis was significantly induced outside, but unaffected inside, the tibial bone by either castration or NVP-AEW541, and the maximum increase (2.7-fold) was obtained by the combined treatment. Proliferation was significantly reduced by NVP-AEW541, independently of growth site, although the maximum decrease (24%) was observed when NVP-AEW541 was combined with castration. Tumor cell IGF-1R immunoreactivity was evaluated in clinical PCa bone metastases (n = 61), and positive staining was observed in most cases (74%). In conclusion, IGF-1R inhibition may be evaluated in combination with ADT in patients with metastatic PCa, or in combination with therapies for the subsequent development of castration-resistant disease, although diverse responses could be anticipated depending on metastasis site.

**Electronic supplementary material:**

The online version of this article (doi:10.1007/s10585-017-9848-8) contains supplementary material, which is available to authorized users.

## Introduction

Prostate cancer (PCa) is one of the most common malignancies among men world-wide. It shows a wide clinical diversity, ranging from slow tumor growth and very low risk of dying from the disease to a highly progressive fatal disease that frequently metastasize to the skeleton [1]. The reason for tumor cell dissemination to the bone marrow and the beneficial growth of tumor cells in the bone environment is not completely understood, but high levels of growth promoting factors, such as the insulin-like growth factors (IGFs), transforming growth factor-β (TGFβ) and bone morphogenic proteins (BMPs) within the bone matrix likely contribute [1, 2]. Numerous studies have pointed at the importance of IGF-1 and its receptor (IGF-1R) during the process of malignant transformation and for stimulation of tumor growth (reviewed in [3]). Convincing evidence has also been presented demonstrating that the IGF-1 system can stimulate tumor cell metastasis to bone. In a mouse model of breast cancer, local bone resorption was shown to stimulate bone metastasis to that site, while disruption of IGF-1R signaling completely abolished this effect [4]. The IGF-1R has been considered a possible therapeutic target in cancer for a long time and many clinical trials have started, and are still ongoing, were IGF-1R signaling is disrupted, either by monoclonal antibodies or by small tyrosine kinase inhibitors given as single agents or in combinations with conventional cancer therapies (http://www.clinicaltrials.gov). Unfortunately, several clinical trials have reported disappointing results with a lack of treatment response in unselected patient cohorts and the development of troublesome side-effects such hyperglycemia (reviewed in [3]). The possibility of giving anti-IGF-1R therapy in combination with standard therapies for advanced disease, however, raises the question if acute therapeutic effects could be potentiated by providing IGF-1R inhibition to selected patients during a limited time-period and, thus, without the risk of introducing adverse effects by long-term IGF-1R inhibition.

The conventional treatment for advanced PCa is androgen deprivation therapy (ADT). In response to surgical castration, proliferation of prostate epithelial cells rapidly declines while apoptosis increases, and the most pronounced effects are seen a few days after treatment in parallel to reduced IGF-1 levels and IGF-1R signaling [5–7]. The magnitude of both IGF-1 reduction and apoptosis induction is less pronounced in malignant compared to non-malignant epithelial cells [5, 6], and we hypothesize that acute effects of castration in PCa and PCa bone metastases may be potentiated by concurrent inhibition of survival signals from IGF-1. To test this principle in vivo, rats with Dunning R3327-G tumor cells inoculated and grown in tibia [8] were treated with the IGF-1R specific tyrosine kinase inhibitor NVP-AEW541 (Novartis) in combination with castration, or by each treatment alone, and acute tumor growth inhibitory effects were compared. The Dunning R3327-G cell line was chosen as model system based on its similarities with PCa in patients; Dunning R3327-G cells are stimulated by androgens but show a limited response to ADT, particularly when growing in a bone environment [8]. In patients, PCa bone metastases primarily induce an osteoblastic, sclerotic bone response [1, 9]. The relevance of using Dunning R3327-G cells to represent PCa bone metastasis growth was therefore evaluated in rat tibia by measuring markers for osteoblast and osteoclast activity. Furthermore, the rationale of using anti-IGF-1R therapy in treatment of PCa patients was evaluated by examining IGF-1R immunoreactivity in clinical bone metastases.

## Methods

### Patients

Bone metastasis samples were obtained from a series of formalin-fixed biopsies collected from patients with PCa operated for metastatic spinal cord compressions or pathologic fractures at Umeå University Hospital (2003–2015). The patient series has been previously described [10, 11] and clinical characteristics are summarized in Supplementary Table 1. Expression of IGF-1R was assessed by immunohistochemistry in 61 biopsies (patients) of which 42 had been previously profiled by whole-genome expression analysis [11] and data was available for functional enrichment analysis, see below. The study was approved by the local ethical review board of Umeå University.

### Cells

Rat prostate Dunning R3327-G tumor cells (94,101,453, ECACC, Salisbury, UK) were maintained in RPMI 1640 + GlutaMAX (Gibco, Life Technologies, Grand Island, NY, US) supplemented with 10% fetal bovine serum (FBS) and 250 nM dexamethasone. The Dunning G cells express the androgen receptor (AR) and are androgen sensitive with low metastatic capacity, according to the original report [12].

### Animal studies

**Fig. 1:**
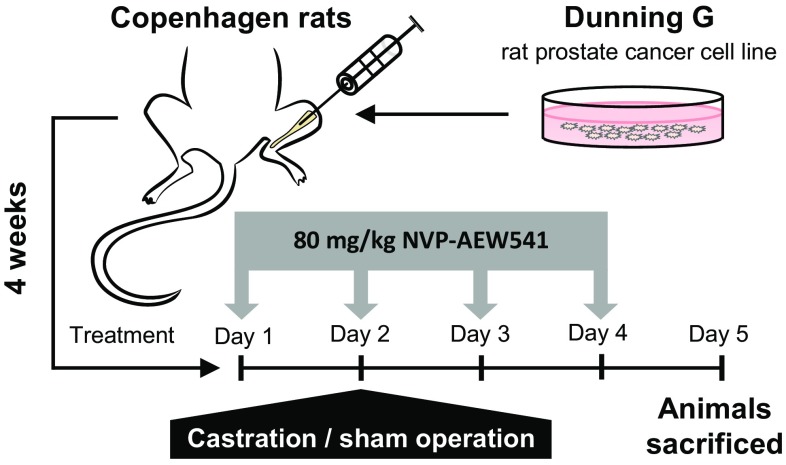
Experimental setup. Dunning G tumor cells were injected into the tibial bone of immune-competent rats (n = 31). Four weeks later 15 rats were administered the anti-IGF-1R therapy NVP-AEW541, p.o. at 80 mg/kg/day, for 4 days. On the 2nd treatment day, 8 of the NVP-AEW541-treated and 8 of the non-treated rats were castrated and the others were sham-operated (n = 15). Animals were sacrificed 3 days later

Intra-tibial injections of Dunning G cells (2 × 10^6^ cells in 20 µl RPMI) were performed in anesthetized male Copenhagen rats (Charles River, bred in-house, n = 31). Four weeks later half of the rats (n = 15) were treated per oral gavage with 80 mg/kg/day of NVP-AEW541 (drug kindly provided and dose recommended [13] by Novartis Pharma, Basel, Switzerland) dissolved in 25 mM L(+)-tartaric acid (Sigma-Aldrich), and the other half with 25 mM L(+)-tartaric acid only. On the second day of treatment, eight of the NVP-AEW541-treated and eight of the control rats were castrated and the remaining rats (n = 15) were sham operated (Fig. 1). At sacrifice, on the fifth day of treatment, rats were injected i.p. with bromodeoxyuridine (BrdU 50 mg/kg; Sigma-Aldrich) and 1 hour later animals were sacrificed. Tumor-containing tibias were dissected and weighed before fixed in 4% paraformaldehyde for 48 h, and subsequently embedded in paraffin. Formalin-fixed tibias were decalcified in formic acid-sodium citrate solution (30 and 15%, respectively) for 48 h and dehydrated before embedded. Six rats were injected with RPMI only and sacrificed 5 weeks later. Animal work was carried out in accordance with protocol approved by the Umeå ethical committee for animal studies (permit number A5-15).

### Cell proliferation in vitro

Dunning G cells (5000 cells/well) were seeded in 100 µl phenol-free RPMI (Gibco) supplemented with 2.5% charcoal stripped FBS (Gibco), in 96-well plates, and allowed to settle overnight. Culture medium was carefully removed and replaced with 100 µl phenol-free RPMI supplemented with 2.5% charcoal stripped FBS and various concentrations of recombinant rat IGF-1 (R&D Systems, Minneapolis, MN, US), dihydrotestosterone (DHT, Sigma-Aldrich, St Louis, MO, US) and NVP-AEW541, at doses previously described [13]. The relative amount of viable tumor cells was measured at baseline, and day 2, 4 and 7 using the Cell Proliferation Kit I (Roche Diagnostics, Bromma, Sweden) according to the manufacturer’s instructions. Cells that were cultured for 7 days received fresh media with supplements at day 4 of the experiment.

### ELISA

Dunning G cells (1.5 × 10^5^) were seeded in six well plates in RPMI 1640 + GlutaMAX (Gibco, Life Technologies, Grand Island, NY, US) supplemented with 10% FBS and 250 nM dexamethasone, and 24 h later medium was replaced with phenol-free αMEM (Gibco) supplemented with 0.1% albumin. After another 24 h, medium was replaced by fresh medium, with or without the addition of 10 nM DHT. Levels of IGF-1 released from Dunning G cells was measured in the culture medium after 72 h using the Mouse/Rat IGF-1 Quantikine ELISA kit (MG100, R&D Systems) according to the manufacturer’s instructions.

### RNA extraction and cDNA synthesis

Total RNA was isolated from tumor cells in culture, as described above, using the RNaqueous kit (Ambion, Life Technologies, Austin, TX, US) according to the manufacturer’s instructions. Samples were DNase treated using TURBO DNase (Ambion), and RNA quality and concentrations measured with the NanoDrop ND-1000 (Nanodrop Technologies, Wilmington DE, US). RNA (500 ng) was reverse transcribed using the First Strand cDNA Synthesis kit (Thermo Fisher Scientific, Waltham, MA, US) according to the manufacturer’s instructions.

### Quantitative real-time polymerase chain reaction

Quantitative real-time PCR analysis of *Igf-1, Igf-1r*, and beta actin (*Actb*) transcripts was performed using TaqMan assays (Rn00710306_m1, Rn00583837, Rn00667869_m1, respectively) the TaqMan Universal PCR Master Mix (Applied Biosystems), and the ABI PRISM 7900 HT Sequence Detection System (Applied Biosystems, Life Technologies, Warrington, UK). Ct values were analyzed with the standard curve method (User Bulletin #2, Applied Biosystems) and normalized to housekeeping gene *Actb*.

### Immunohistochemistry

Tissue sections were deparaffinized in xylene and rehydrated through graded ethanol. For histological examinations, clinical sections were stained in hematoxylin-eosin and rat tibia sections in van Gieson. Human IGF-1R was detected after antigen retrieval in TRIS/EDTA, pH 9, by incubation with the IGF-1R antibody (AF-305-NA, R&D Systems, diluted 1:25) at 4 °C overnight followed by the Goat-HRP-Polymer detection kit (GHP516G, Biocare Medical) and DAB as chromogen. Rat IGF-1R was detected using a similar protocol but with primary antibody incubation performed in room temperature for 2 h followed by a secondary horse anti-goat antibody (1:200, BA-9500) and the ABC detection kit (Vector laboratories, Burlingame, CA). Other immunostainings in rat tissues were performed using the Benchmark Ultra system (Ventana) and the ultraView Universal DAB Detection Kit (760–500, Ventana). Sections were incubated with antibodies for AR (pG21, 06-680, Millipore, diluted 1:100), IGF-1 (05-172, Millipore, diluted 1:50), and BrdU (347,580, BD Biosciences, diluted 1:200) after antigen retrieval in the CC1 buffer (plus protease-1, 4 min, for BrdU, Ventana) or with antibodies for tartrate-resistant acid phosphatase (TRAP, MABF96, Millipore, diluted 1:400) and runt-related transcription factor 2 (RUNX2, ab81357, Abcam, diluted 1:100) after antigen retrieval in the CC2 buffer (Ventana). Activated caspase-3 was detected by an antibody (9661L, Cell signaling, diluted 1:200) recognizing its cleaved form (Asp175) and the ABC detection kit (Vector) after boiling in EDTA (pH 8) for 45 min followed by 0.2% pepsin in 37 °C for 5 min. Negative control sections were prepared by performing immunostaining procedures without adding primary antibodies.

**Fig. 2:**
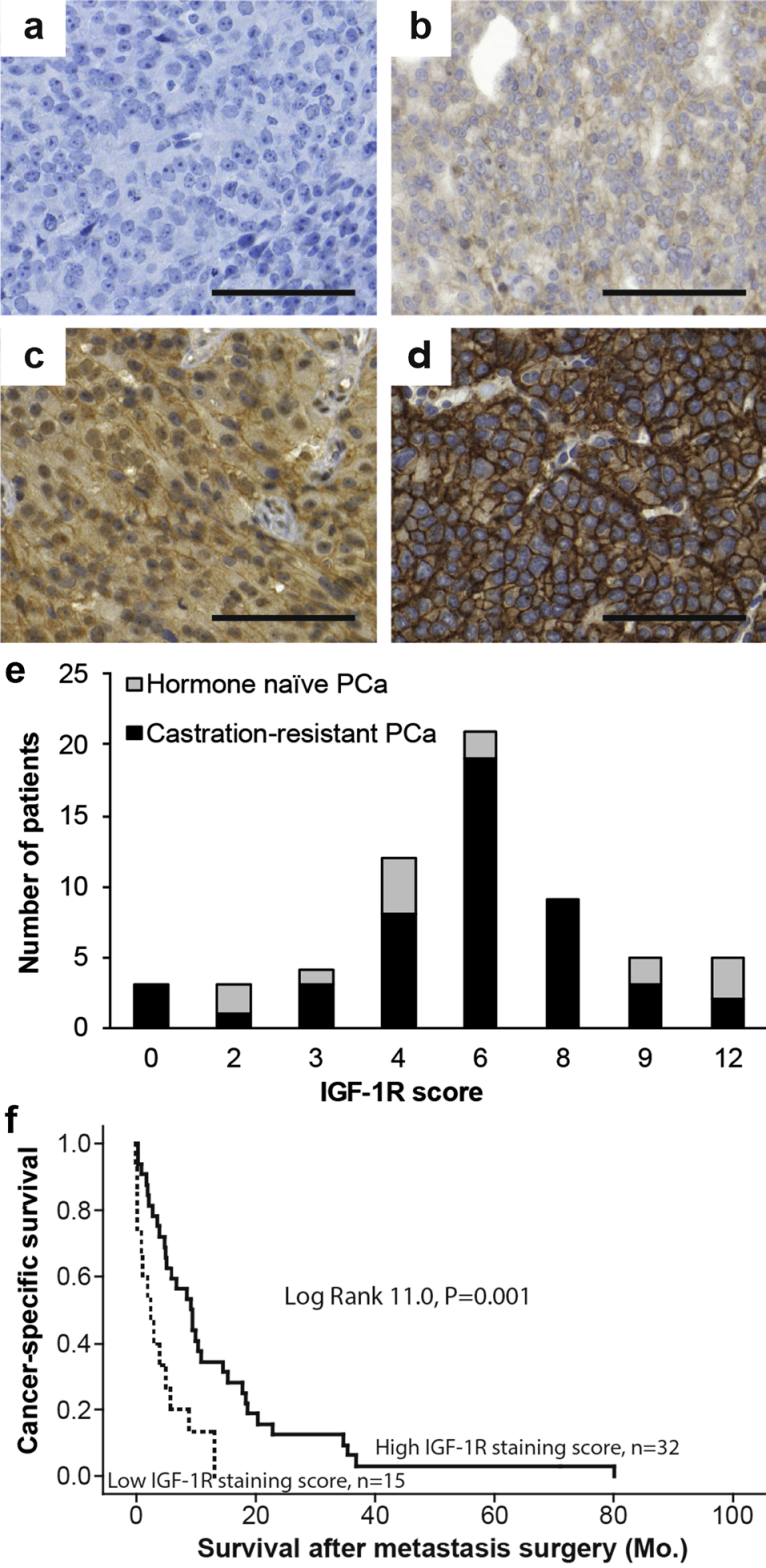
Immunohistochemical staining of IGF-1R in human prostate cancer bone metastases. A total IGF-1R score was calculated by multiplying tumor cell staining intensity (0–3) with the fraction of stained cells (0–4) for each metastasis. Sections show representative staining of different intensity, **a** no staining (0), **b** weak (1), **c** moderate (2), and **d** intense (3) staining (*bar* indicates 100 µm).** e** A total IGF-1R score was determined in bone metastases from 14 hormone-naïve and 47 castration-resistant prostate cancer patients. **f** Cancer-specific survival of castration-resistant patients in relation to the total IGF-1R score in their bone metastases, dichotomized as high (6–12, i.e. above median) or low (0–4, i.e. below median)

The IGF-1R staining in clinical metastases was quantified by scoring the intensity (0 = no staining, 1 = weak, 2 = moderate, 3 = intense staining, Fig. 2a–d) and the percentage of tumor cells stained (1 = 0–25%, 2 = 26–50%, 3 = 51–75%, 4 = 76–100%). A combined staining score, ranging from 0 to 12, was then calculated by multiplying intensity with distribution. The IGF-1 and IGF-1R stainings in Dunning R3327-G rat tumors were assessed by scoring the intensity, as described above for the clinical metastases. The proliferation rate (fraction of BrdU-positive tumor cells) and the apoptotic rate (fraction of tumor cells positive for activated caspase-3) in the Dunning tumors were scored by evaluating 1000 and 2000 cells, respectively, per sample, in cells situated inside or outside the tibial bone marrow cavity. Trabecular bone surface adjacent to TRAP-positive cells and total trabecular bone surface was measured using the ImageJ 1.50i software in a defined area within the tibial bone marrow cavity. To distinguish TRAP-positive osteoclasts from TRAP-positive chondroclasts all measurements were made no less than 250 µm from the hypertrophic mineralizing zone.

### Multivariate modeling and functional enrichment analysis

Multivariate modelling by means of orthogonal projections to latent structures (OPLS) was used to extract systematic variation in whole-genome expression data from human PCa bone metastasis samples previously analysed [11], in relation to corresponding IGF-1R IHC score in each sample. OPLS-DA was performed in SIMCA version 14.0 (MKS Umetrics AB, Umeå, Sweden) on mean centred and unit variance scaled gene expression data and validated by cross-validation. The influence of individual genes on the OPLS-model was calculated as the variable influence on the projections (VIP) to latent structures [14], and VIP values over 2 were considered relevant and subjected to gene set enrichment analysis.

Enrichment analysis was performed using the MetaCore software (GeneGo, Thomson Reuters) in order to identify enriched process networks in the data. The significance of the association between the list of molecules in the data (gene transcripts with VIP > 2, see above) and enrichment of process networks was assessed by: (i) the ratio of molecules in the data that mapped to a specific network in relation to the total number of molecules included in the network and (ii) the false discovery rate when applying the Fisher’s Exact test to determine the probability that the relationship between the molecules in the data set and the networks was explained by chance.

### Statistical analysis

Groups were compared using the independent samples t-test or paired samples t-test, respectively. Kaplan–Meier survival analysis was performed with death due to PCa as event and follow-up time as the time between metastasis surgery and the latest follow-up examination. Statistical analyses were performed using the Statistical Package for the Social Sciences, SPSS 24.0 software (SPSS, Inc, Chicago, USA).

## Results

### Expression of IGF-1R in the majority of clinical PCa bone metastases

To evaluate the fraction of patients who may benefit from receiving anti-IGF-1R therapy in combination with ADT, or at later stages in combination with therapies given for castration-resistant prostate cancer (CRPCa), a series of bone metastasis biopsies from treatment-naïve (n = 14) and castration-resistant (n = 47) patients were evaluated for IGF-1R immunoreactivity. Moderate to intense IGF-1R staining was found in 71% (10/14) of the treatment-naïve and 74% (35/47) of the CRPCa bone metastases (Fig. 2a–e). Patients with CRPCa and IGF-1R staining scores of 0–4, i.e. scores below median showed shorter cancer-specific survival than patients with staining scores of 6 and above; median survival was 2.5 versus 9.3 months (log-rank 11, *P* = 0.001, n = 15 vs. 32, Fig. 2f). No other relation was observed between IGF-1R immunoreactivity and the clinical variables in supplementary Table 1.

Multivariate modeling of whole-genome expression profiles, available for 42 clinical metastases, in relation to their corresponding IGF-1R immunoreactivity scores identified 133 gene transcript with positive and 397 gene transcripts with negative correlations (VIP > 2, Supplementary Table 2). Gene products with negative correlations to IGF-1R protein expression were enriched for process networks involving immune cells, while positively correlated transcripts were enriched for translation and cell cycle associated processes (Supplementary Table 3). In other words, bone metastases with high IGF-1R immunoreactivity were suspected to have low immune cell infiltration and high proliferation, similar to what we recently reported for AR driven CRPCa bone metastases [10, 11], supporting our strategy of strengthened ADT effects by concurrent anti-IGF-1R therapy.

### Dunning G cells are stimulated by IGF-1 and DHT in vitro

**Fig. 3:**
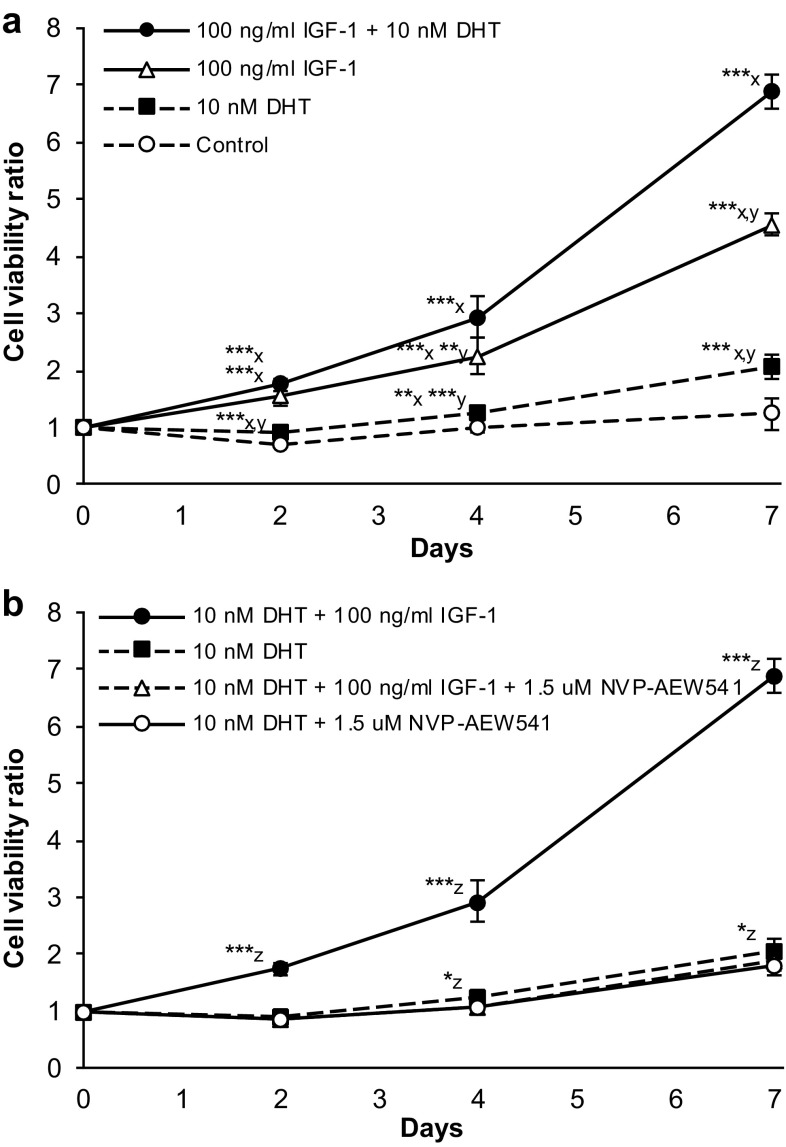
Tumor cell viability after stimulation with IGF-1 and DHT and inhibition with NVP-AEW541. Dunning G cell viability was determined after 2, 4 and 7 days of **a** stimulation with 100 ng/ml IGF-1 and/or 10 nM DHT and **b** additional inhibition with 1.5 µM NVP-AEW541. The stimulatory effect of IGF-1 on Dunning G cells was completely eliminated by NVP-AEW541. Data is presented as fold change in mean absorbance ± SE compared to day 0, and each point is represented by six replicates. Statistical significance is assessed at each time point and marked with x when compared to control cells, y when compared to Dunning G cells treated with 100 ng/ml IGF-1 and 10 nM DHT, and z when compared to Dunning G cells treated with 10 nM DHT (*P < 0.05, **P < 0.01, ***P < 0.001)

The Dunning R3327-G cells expressed IGF-1 and the IGF-1R receptor when growing in culture, and the IGF-1 secretion was positively stimulated by DHT (Supplementary Figure 1). Growth of the Dunning G cells was significantly stimulated by DHT and further increased by the addition of recombinant rat IGF-1 at different concentrations over a time-period of 7 days (Fig. 3a, Suppl. Figure 2). A 2.3-fold and 4.6-fold increase in tumor cell viability was seen after 4 respectively 7 days in culture with 100 ng/ml of IGF-1. To confirm that the IGF-1 induced increase in viability could be blocked by the IGF-1R inhibitor NVP-AEW541, cells were stimulated with DHT and 100 ng/ml IGF-1 in the presence of various concentrations of NVP-AEW541. At 1.5 µM NVP-AEW541, the lowest concentration tested, the increase in cell viability induced by 100 ng/ml IGF-1 was normalized (Fig. 3b). Cells treated with 3 µM NVP-AEW541 also had a viability that closely resembled control cells not receiving any IGF-1, while 6 µM of NVP-AEW541 were toxic to the Dunning G cells (results not shown).

### Dunning G cells induce bone remodeling in rat tibia

**Fig. 4:**
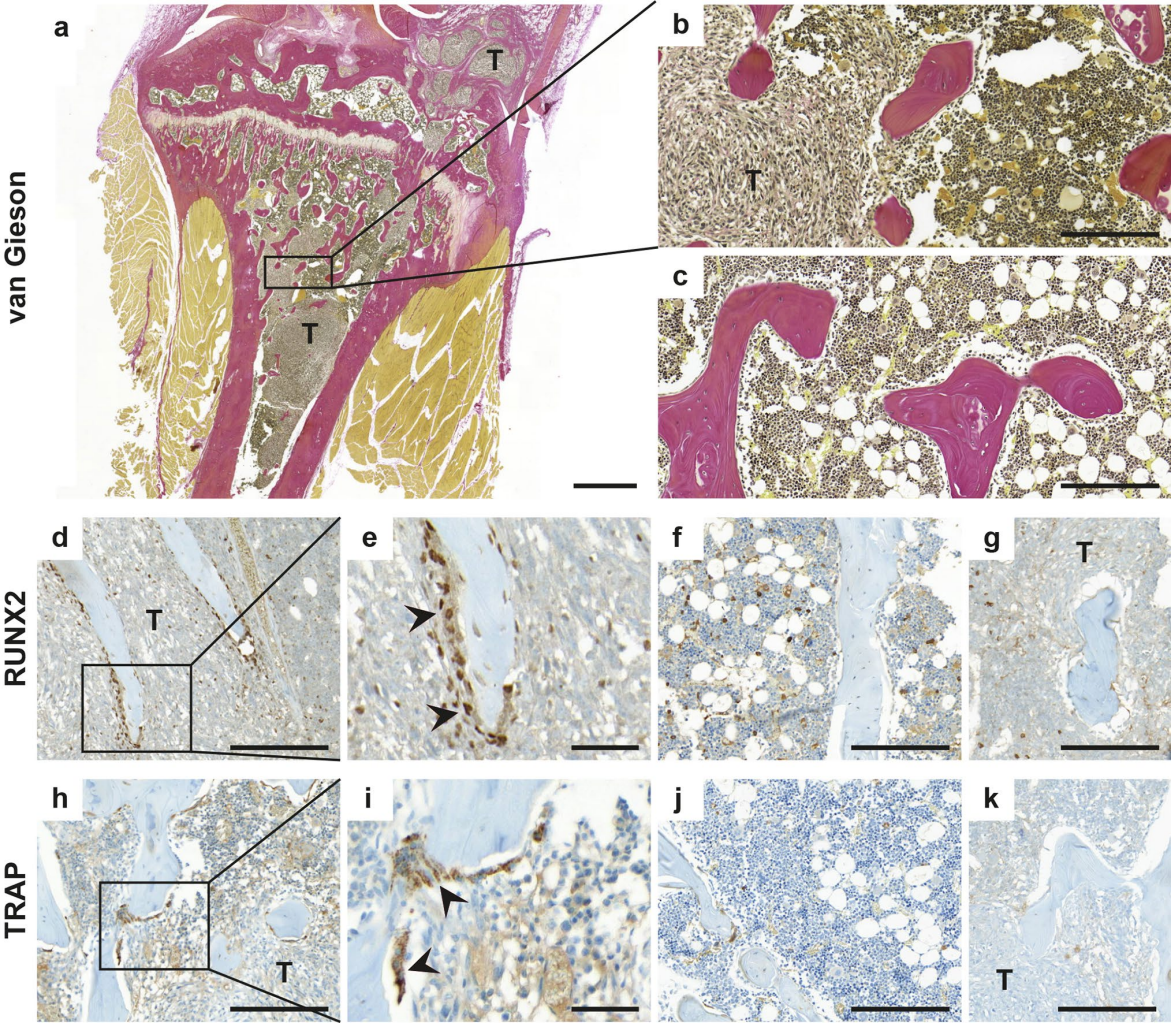
Increased bone remodeling activity in rat tibia after tumor cell injection. Representative sections of tibial bones with (**a**–**b**,** d**–**e**,** g**–**i**,** k**) or without (**c**,** f**,** j**) growth of Dunning G tumor cells, about 5 weeks after injection. **a** Section showing tumor cells (T) located both in the bone marrow cavity and outside of the tibial bone, in the knee joint area. Staining of osteoblast marker RUNX2 (**d**–**f**) and osteoclast marker TRAP (**h**–**j**) indicating frequently more activated osteoblasts and osteoclasts in bones with than without injected Dunning G tumors. Negative control stainings are represented by **g, k**. *Bar* in **a** indicates 1000 µm, *bars* in (**b**–**d, f**–**h, j**–**k**) indicates 200 µm, and *bars* in (**e, i**) indicates 50 µm

To evaluate how Dunning G tumor cells effected bone cell remodeling in rat tibia, osteoblast and osteoclast activity were compared about 5 weeks after injection of tumor cells (n = 8) or RPMI only (n = 6). When Dunning R3327-G cells were growing in the rat tibia (Fig. 4a–b), cells exhibiting the osteoblast-specific transcription factor RUNX2 were clearly detected along the trabecular bone surfaces, while RUNX2-positive cells were scarcely seen in control tibias (Fig. 4d–f). Tumor-bearing rats also had a 4.6-fold increase in trabecular bone surfaces lined by TRAP-positive osteoclasts (36.6 ± 21.6 × 10^−3^ vs. 7.9 ± 6.5 × 10^−3^ TRAP-positive surface/total bone surface, P = 0.009, n = 14, Fig. 4h–j). No significant difference in trabecular bone volume between tumor-containing and tumor-free tibias was observed, as determined from the total bone surface per evaluated area in the bone marrow (4.2 ± 1.1 × 10^−3^ vs. 3.5 ± 0.8 × 10^−3^ mm/mm^2^, P = 0.27, n = 14).

Taken together, those results indicate that Dunning R3327-G cells induce bone remodeling in the rat tibia, through activation of both osteoblasts and osteoclasts. A longer tumor growth time in tibia may have been needed to fully reveal potential effects of the Dunning cells on bone mass.

### Effects of castration and NVP-AEW541 on Dunning G tumor cells depend on the microenvironment

In order to evaluate PCa cell response to anti-IGF-1R treatment in combination with castration, Dunning R3327-G cells were injected into the tibia of male Copenhagen rats and treated about 4 weeks later (Fig. 1). Tumor cell growth was observed in the bone marrow cavity, but occasionally also outside the bone in the knee joint cavity and in muscle tissue (Fig. 4a). Different tumor cell microenvironments could have potentially different influence on tumor cell response to therapy [8], and we therefore decided to evaluate therapeutic effects in tumor cells situated within the tibial bone marrow cavity separately from therapeutic effects in tumor cells situated elsewhere.

**Fig. 5:**
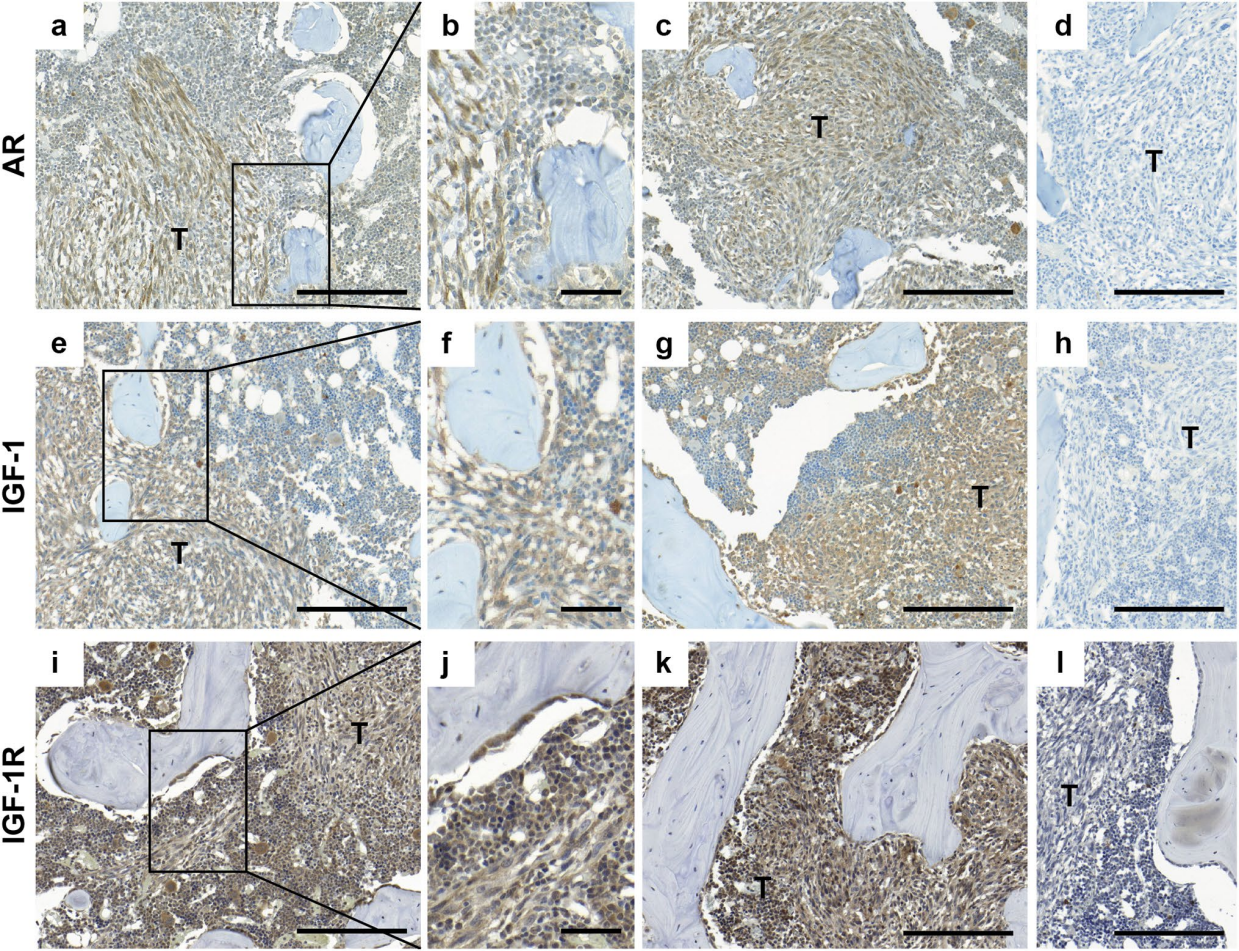
Immunohistochemical staining of AR, IGF-1, and IGF-1R in Dunning G cells growing in rat tibia. Representative pictures showing immunostaining of the AR (**a**–**c**), IGF-1 (**e**–**g**), and IGF-1R (**i**–**k**) in Dunning G tumors located within the tibial bone marrow cavity of non-castrated (**a, b, e, f, i, j**) and castrated (**c, g, k**) rats. Negative controls stainings are represented by (**d, h, l**). *Bar* in (**a, c**-**e, g**-**i, k**-**l**) indicates 200 µm and *bar* in (b, f, j) 50 µm

Positive immunoreactivity for AR, IGF-1 and IGF-1R was seen in Dunning tumor cells *in vivo*, with no differences in staining intensities observed between tumor cells growing inside or outside the bone marrow cavity (Fig. 5 and data not shown). Positive IGF-1 and IGF-1R immunoreactivities were seen also in the tumor micro-environment, including cells lining the bone surfaces, likely osteoblasts (Fig. 5f, g). Outside tibia, strong immunoreactivity for AR, IGF-1, and IGF-1R were seen in both skeletal and smooth muscles (data not shown). The AR, IGF-1, and IGF-1R staining were largely unaffected by castration (Fig. 5).

**Fig. 6:**
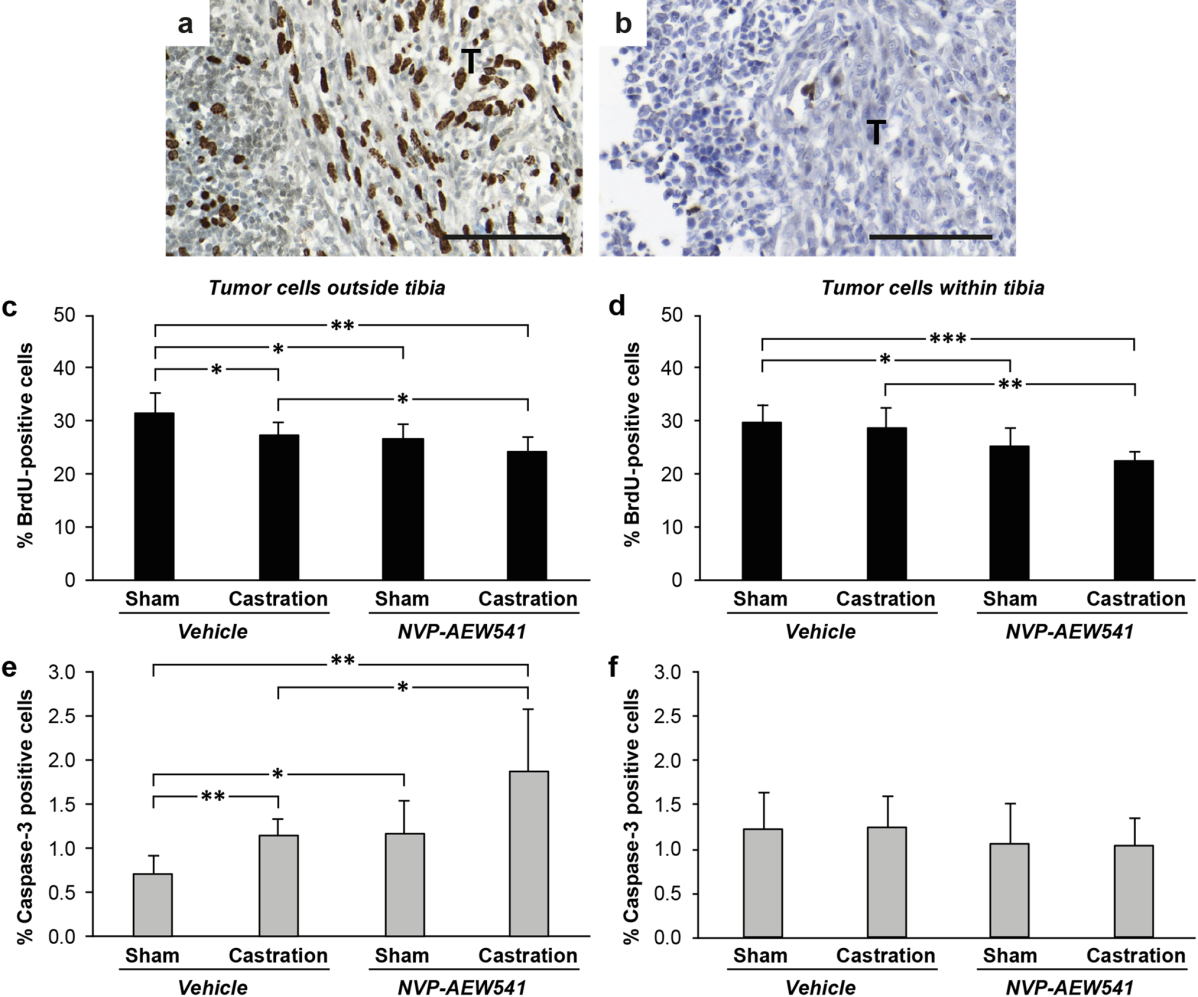
Proliferation and apoptosis in tumor cells following castration and/or NVP-AEW541 treatment in rats. Sections show representative stainings of **a** proliferating tumor cells (BrdU) and **b** apoptotic tumor cells (caspase-3) in bone marrow of untreated animals. *Bar* indicates 100 µm. Proliferation decreased in Dunning G cells growing both outside (**c**) and inside the tibial bone marrow cavity (**d**). Dunning G cell apoptosis within the bone marrow was not affected by any of the treatments (**f**), while apoptosis increased outside the tibial bone by each treatment given (**e**). Each *bar* represents means ± SD; n = 5–8 animals (*P < 0.05, **P < 0.01, ***P < 0.001)

Effects of castration and/or NVP-AEW541 treatment were studied by examining tumor cell proliferation and apoptosis rates 3 days after castration, at the time-point where maximum castration-induced effects have been reported in human and rat prostates as well as in Dunning G tumors [5, 15–17]. Proliferating tumor cells were detected by immunostaining of incorporated BrdU and apoptotic cells by positive staining for activated Caspase-3 (Fig. 6a, b). Castration significantly reduced the mean proliferation rate by 13% and induced the mean apoptotic rate 1.6-fold, in tumor cells growing outside the bone marrow. Those effects were significantly potentiated by anti-IGF-1R (NVP-AEW541) treatment, resulting in 0.77- and 2.7-fold changes, respectively, in comparison to basal levels (Fig. 6c, e). In contrast, neither proliferation nor apoptosis was affected by castration in tumor cells growing inside the bone marrow cavity (Fig. 6d, f). The basal apoptotic rate was however 1.8-times higher in tumor cells growing inside compared to outside the bone marrow cavity (*P* = 0.006, Fig. 6e, f), and was not further induced by any of the therapies given. Importantly, the anti-IGF-1R treatment significantly reduced tumor cell proliferation independently of growth site, with the maximum decrease (24%) observed within the bone marrow cavity when NVP-AEW541 was given in combination with castration (Fig. 6c-d).

## Discussion

The present study shows the suitability of using the rat Dunning R3327-G cell line in experimental studies of PCa growth in bone. The Dunning R3327-G cells are stimulated by DHT and IGF-1, and induce bone remodeling by increased osteoclast and osteoblast activity when inoculated into the rat tibia. This biology would result in a positive bone scan, associated with the sclerotic phenotype seen in most cases of clinical PC bone metastases. Accordingly, induced expression of both osteoblast and osteoclast activity markers is observed in most bone metastases examined from PC patients (own unpublished data) and elevated serum levels of bone resorption markers are found in patients with sclerotic bone metastases (reviewed in [9]). Furthermore, the present study shows that inhibition of pro-survival IGF-1R signaling has the potential to enhance acute therapeutic effects of castration in PCa, but importantly also clearly demonstrates that tumor cell response to castration and anti-IGF-1R treatment may differ depending on the tumor cell microenvironment. Specifically, the Dunning R3327-G cells show induced apoptotic and reduced proliferation rates in response to castration only when growing outside the tibial bone marrow cavity, and not when situated within the bone marrow cavity. In contrast, the anti-IGF-1R treatment seems to decrease the proliferation rate of Dunning R3327-G cells irrespectively of growth site, but likewise castration to induce apoptosis only in tumor cells growing outside the bone marrow cavity. As the majority of clinical bone metastases examined expressed considerable levels of IGF-1R it is tempting to speculate that IGF-1R inhibition would have beneficial therapeutic effects if given to the corresponding patients, although the apoptotic response may be larger in extra-skeletal metastases than in metastatic cells lying within the bone-marrow microenvironment.

The importance of the microenvironment for castration-induced tumor cell apoptosis has been previously demonstrated for Dunning G cells [8] and also for Dunning AT-1 cells [18]. When orthotopically grown in the rat ventral prostate (VP), Dunning G cells showed a pronounced apoptotic response to castration, while in line with the current results no response was observed in Dunning G cells growing within the tibia [8]. Castration-induced apoptosis was observed also in AR-negative Dunning AT-1 cells growing in the rat VP, probably mediated through castration-induced effects in the androgen-regulated prostate stroma, i.e. through reduced vascular blood flow and reduced IGF-1 expression [6, 7, 18]. The mechanism behind the castration-induced effects seen in Dunning G cells growing outside the tibial bone as well as the lack of response within the bone marrow is not clear. It is not known if cells in the microenvironment outside tibia, i.e. in the knee joint and in the surrounding skeletal muscle, are androgen-regulated in a similar way as the VP stroma or if effects of castration on Dunning G cells growing outside the bone marrow cavity are mediated solely by disrupted AR signaling in the tumor cells. The lack of castration effects in Dunning G tumor cells within the bone marrow cavity may be explained by the presence of other pro-survival and/or anti-apoptotic factors than IGF-1. The rat bone marrow contains low levels of androgens in comparison to the rat VP and rat plasma, and is also more hypoxic [8]. Low androgen levels and hypoxia probably contribute to the high basal apoptotic rate observed in Dunning G cells growing inside the bone marrow. Most likely, hypoxia also drive tumor growth and progression by expression of hypoxia-induced, pro-survival, metabolic and angiogenic factors in tumor cells and in the bone microenvironment [19]. Moreover, the robust increase of RUNX2-positive osteoblasts observed in rat tibia in the vicinity of Dunning G cells indicates tumor cell dependent induction of osteoblast differentiation and increased bone formation. This did not, however, result in an increased bone mass most likely due to the parallel osteoclast formation and activation. Induced osteoblast differentiation will result not only in bone forming osteoblasts but also in an enhanced number of late osteoblasts differentiating into osteocytes, cells that are the main producer of RANKL [20]. It is, therefore, not surprising that induction of osteoblast differentiation by tumor cells also results in increased formation of osteoclasts. These observations are in line with the seed-and-soil hypothesis [21] postulating that increased osteoclast activation and release of growth promoting factors from the bone matrix, including IGF-1, are important events favoring tumor growth in the skeleton [22]. The low androgen level and low oxygen pressure in the bone marrow may be the reasons to why the already high basal apoptotic level of tumor cells within the bone marrow is not further enhanced by castration, and tumor-induced activation of osteoblasts may result in production/release of pro-survival factors counteracting therapy-induced tumor cell apoptosis.

Several preclinical studies have indicated the importance of the IGF axis for PCa progression and provided the basis for clinical trials with the IGF-1R as an anti-cancer target, alone or in combination with conventional therapies (recently reviewed in [23]). Reciprocal activation has been observed between the IGF-1R and the AR signaling pathways, supporting the rationale of simultaneous inhibition in cancer treatment [24, 25]. In the LuCaP 35 xenograft model, treatment with a monoclonal anti-IGF-1R antibody (A12) prevented AR re-activation after castration and markedly prolonged time until castration-resistance [26]. This promising preclinical study was followed by a phase II clinical study where ADT was combined with Cixutumumab (A12) for treatment of patients with hormone-naïve metastatic PCa [27]. The treatment was well tolerated but the primary endpoint; increased frequency of undetectable PSA levels after 28 weeks, was not reached. Patients in this trial need to be continuously followed for time to castration-resistance and overall survival. Based on the results in the current study, a sub-analysis of outcome in relation to metastatic site could be argued. Furthermore, a sub-analysis of outcome in relation to metastasis IGF-1R expression would have been preferred.

The inverse relation between IGF-1R expression and immune cell functions, observed here in the clinical bone metastases, suggests that long-term IGF-1R inhibition may have the potential to stimulate endogenous T cells activity and thus tumor immunosurveillance. Previous experimental studies have shown activation of host immune response to tumor cells after IGF-1R knock-down using anti-sense based strategies [28–30]. This justifies further examination of a possible connection between tumor cell expression of IGF-1R and tumor cell immune-evasion. In a carefully designed preclinical study, siRNA was used to down-regulate IGF-1R expression in a murine breast cancer cell line and found to decrease proliferation, but also to induce secretion of pro-inflammatory cytokines and to slow down tumor growth in syngenic mice partly by activating anti-tumor immune cell responses [31]. Not to forget, however, CRPCa patients with low metastatic IGF-1R expression had a shorter cancer-specific survival than patients with high IGF-1R expression, despite their suggested increase in immune cell activity. This highlights the need for novel therapies also for this sub-group of most likely non-AR driven metastases [11], and one strategy could be by strengthening their endogenous immune response by inhibition of myeloid derived suppressor cells or of co-inhibitory systems; PD1/PD-L1 or CTLA4 [32].

To summarize, our results open up a therapeutic window were IGF-1R inhibition could be given to patients with metastatic PCa during a short period of time in order to boost acute anti-proliferative and pro-apoptotic effects of ADT or other therapies given for CRPCa, although with the possibility that response may differ depending on tumor cell microenvironment and thus metastatic site. Conclusions from the current study are limited by the use of one tumor cell line only, and findings thus warrant further investigations in complementary model systems for PCa. If short-term term anti-IGF-1R treatment could give prolonged inhibitory effects on tumor growth, by enhancing immune cell recognition and eradication of tumor cells and if those effects are better induced by anti-IGF-1R antibodies than by tyrosine kinase inhibitors, or by nucleic acid strategies only, also need to be further examined.

## Electronic supplementary material

Below is the link to the electronic supplementary material.

Supplementary Figure 1 Relative levels of *Igf-1* and *Igf-1r* mRNA (a) and secreted levels of IGF-1 protein (b) from Dunning G cells cultured with or without the addition of 10 nM DHT. Stimulation with DHT induces increased *Igf-1* expression and an increased release of IGF-1 protein from the Dunning G cells. Data is expressed in relation to Dunning G cells without DHT (set to 1) and each bar represents means ± SD of 6 replicates. Statistically significant changes induced by co-culture are marked with asterisks (* P < 0.05; *** P < 0.001)

Supplementary Figure 2 Tumor cell viability after stimulation with IGF-1 and DHT. Stimulation of Dunning G cells by 10 nM DHT in combination with (a) 1 ng/ml IGF-1 or (b) 10 ng/ml IGF-1. Data is presented as fold change in mean absorbance ± SE compared to day 0, and each point is represented by 6 replicates. Statistical significance is assessed at each time point and marked with x when compared to control cells, and y when compared to Dunning G cells treated with 10 nM DHT and 1 or 10 ng/ml IGF-1 (*P < 0.05, **P < 0.01, ***P < 0.001)

Supplementary Table 1

Supplementary Table 2

Supplementary Table 3

## Abbreviations

*Actb*: Beta actin
ADT: Androgen deprivation therapy
AR: Androgen receptor
BMPs: Bone morphogenic proteins
CRPCa: Castration-resistant prostate cancer
DHT: Dihydrotestosterone
IGF-1: Insulin-like growth factor 1
IGF-1R: Insulin-like growth factor 1 receptor
OPLS: Orthogonal projections to latent structures
PCa: Prostate cancer
RUNX2: Runt-related transcription factor 2
TGFβ: Transforming growth factor-β
TRAP: Tartrate-resistant acid phosphatase
VIP: Variable influence on projection
VP: Ventral prostate

## References

[1] Weilbaecher KN, Guise TA, McCauley LK (2011). Cancer to bone: a fatal attraction. Nat Rev Cancer.

[2] Hauschka PV, Mavrakos AE, Iafrati MD, Doleman SE, Klagsbrun M (1986). Growth factors in bone matrix. Isolation of multiple types by affinity chromatography on heparin-Sepharose. J Biol Chem.

[3] Pollak M (2012). The insulin and insulin-like growth factor receptor family in neoplasia: an update. Nat Rev Cancer.

[4] Hiraga T, Myoui A, Hashimoto N, Sasaki A, Hata K, Morita Y, Yoshikawa H, Rosen CJ, Mundy GR, Yoneda T (2012). Bone-derived IGF mediates crosstalk between bone and breast cancer cells in bony metastases. Cancer Res.

[5] Ohlson N, Wikström P, Stattin P, Bergh A (2005). Cell proliferation and apoptosis in prostate tumors and adjacent non-malignant prostate tissue in patients at different time-points after castration treatment. Prostate.

[6] Ohlson N, Bergh A, Stattin P, Wikström P (2007). Castration-induced epithelial cell death in human prostate tissue is related to locally reduced IGF-1 levels. Prostate.

[7] Ohlson N, Bergh A, Persson ML, Wikström P (2006). Castration rapidly decreases local insulin-like growth factor-I levels and inhibits its effects in the ventral prostate in mice. Prostate.

[8] Bergström SH, Rudolfsson SH, Bergh A (2016). Rat prostate tumor cells progress in the bone microenvironment to a highly aggressive phenotype. Neoplasia.

[9] Buijs JT, van der Pluijm G (2009). Osteotropic cancers: from primary tumor to bone. Cancer Lett.

[10] Crnalic S, Hörnberg E, Wikström P, Lerner UH, Tieva Å, Svensson O, Widmark A, Bergh A (2010). Nuclear androgen receptor staining in bone metastases is related to a poor outcome in prostate cancer patients. Endocr Relat Cancer.

[11] Ylitalo EB, Thysell E, Jernberg E, Lundholm M, Crnalic S, Egevad L, Stattin P, Widmark A, Bergh A, Wikström P (2016). Subgroups of castration-resistant prostate cancer bone metastases defined through an inverse relationship between androgen receptor activity and immune response. Eur Urol.

[12] Isaacs JT, Isaacs WB, Feitz WF, Scheres J (1986). Establishment and characterization of seven Dunning rat prostatic cancer cell lines and their use in developing methods for predicting metastatic abilities of prostatic cancers. Prostate.

[13] García-Echeverría C, Pearson MA, Marti A, Meyer T, Mestan J, Zimmermann J, Gao J, Brueggen J, Capraro H-G, Cozens R, Evans DB, Fabbro D, Furet P, Porta DG, Liebetanz J, Martiny-Baron G, Ruetz S, Hofmann F (2004). In vivo antitumor activity of NVP-AEW541-A novel, potent, and selective inhibitor of the IGF-IR kinase. Cancer Cell.

[14] Eriksson L, Johansson E, Kettaneh-Wold N, Trygg J, Wikström C, Wold S (2006). Multi- and megavariate data analysis. Metabonomics in toxicity assessment.

[15] Westin P, Bergh A, Damber JE (1993). Castration rapidly results in a major reduction in epithelial cell numbers in the rat prostate, but not in the highly differentiated Dunning R3327 prostatic adenocarcinoma. Prostate.

[16] Pollack A, Lim Joon D, Wu CS, Sikes C, Hasegawa M, Terry NHA, White RA, Zagars GK, Meistrich ML (1997). Quiescence in R3327-G Rat prostate tumors after androgen ablation. Cancer Res.

[17] Joon DL, Hasegawa M, Sikes C, Khoo VS, Terry NHAA, Zagars GK, Meistrich ML, Pollack A, Lim Joon D, Hasegawa M, Sikes C, Khoo VS, Terry NHAA, Zagars GK, Meistrich ML, Pollack A (1997). Supraadditive apoptotic response of R3327-G rat prostate tumors to androgen ablation and radiation. Int J Radiat Oncol Biol Phys.

[18] Halin S, Hammarsten P, Wikström P, Bergh A (2007). Androgen-insensitive prostate cancer cells transiently respond to castration treatment when growing in an androgen-dependent prostate environment. Prostate.

[19] Semenza G (2016). The hypoxic tumor microenvironment: a driving force for breast cancer progression. Biochim Biophys Acta - Mol. Cell Res.

[20] Xiong J, Onal M, Jilka RL, Weinstein RS, Manolagas SC, O’Brien Ca (2011). Matrix-embedded cells control osteoclast formation. Nat Med.

[21] Langley RR, Fidler IJ (2011). The seed and soil hypothesis revisited-The role of tumor-stroma interactions in metastasis to different organs. Int J Cancer.

[22] Roodman GD (2012). Genes associate with abnormal bone cell activity in bone metastasis. Cancer Metastasis Rev.

[23] Heidegger I, Massoner P, Sampson N, Klocker H (2015). The insulin-like growth factor (IGF) axis as an anticancer target in prostate cancer. Cancer Lett.

[24] Pandini G, Mineo R, Frasca F, Roberts CT, Marcelli M, Vigneri R, Belfiore A (2005). Androgens up-regulate the insulin-like growth factor-I receptor in prostate cancer cells. Cancer Res.

[25] Culig Z, Hobisch A, Cronauer MV, Radmayr C, Trapman J, Hittmair A, Bartsch G, Klocker H (1994). Androgen receptor activation in prostatic tumor cell lines by insulin-like growth factor-I, keratinocyte growth factor, and epidermal growth factor. Cancer Res.

[26] Plymate SR, Haugk K, Coleman I, Woodke L, Vessella R, Nelson P, Montgomery RB, Ludwig DL, Wu JD (2007). An antibody targeting the type I insulin-like growth factor receptor enhances the castration-induced response in androgen-dependent prostate cancer. Clin Cancer Res.

[27] Yu EY, Li H, Higano CS, Agarwal N, Pal SK, Alva A, Heath EI, Lam ET, Gupta S, Lilly MB, Inoue Y, Chi KN, Vogelzang NJ, Quinn DI, Cheng HH, Plymate SR, Hussain M, Tangen CM, Thompson IM (2015). SWOG S0925: a randomized phase II study of androgen deprivation combined with cixutumumab versus androgen deprivation alone in patients with new metastatic hormone-sensitive prostate cancer. J Clin Oncol.

[28] Resnicoff M, Li W, Basak S, Herlyn D, Baserga R, Rubin R (1996). Inhibition of rat C6 glioblastoma tumor growth by expression of insulin-like growth factor I receptor antisense mRNA. Cancer Immunol Immunother.

[29] Liu X, Turbyville T, Fritz A, Whitesell L (1998). Inhibition of insulin-like growth factor I receptor expression in neuroblastoma cells induces the regression of established tumors in mice. Cancer Res.

[30] Schillaci R, Salatino M, Cassataro J, Proietti CJ, Giambartolomei GH, Rivas MA, Carnevale RP, Charreau EH, Elizalde PV (2006). Immunization with murine breast cancer cells treated with antisense oligodeoxynucleotides to type I insulin-like growth factor receptor induced an antitumoral effect mediated by a CD8^+^ response involving Fas/Fas ligand cytotoxic pathway. J Immunol.

[31] Durfort T, Tkach M, Meschaninova MI, Rivas MA, Elizalde PV, Venyaminova AG, Schillaci R, François JC (2012). Small interfering RNA targeted to IGF-IR delays tumor growth and induces proinflammatory cytokines in a mouse breast cancer model. PLoS One.

[32] Saad F, Miller K (2015). Current and emerging immunotherapies for castration-resistant prostate cancer. Urology.

