# Supplementary material for: Inhibition of the insulin-like growth factor-1 receptor potentiates acute effects of castration in a rat model for prostate cancer growth in bone
Source: Clin Exp Metastasis. 2017 Apr 26;34(3):261–71. doi: 10.1007/s10585-017-9848-8 (PMC5442252; doi:10.1007/s10585-017-9848-8)
Supplement: Supplementary file 3 — Supplementary Table 1 [file 10585_2017_9848_MOESM3_ESM.pdf]

Supplementary Table 1 Clinical characteristics of patients analyzed for IGF1R immunohistochemistry in bone metastases obtained at surgery for metastatic spinal cord compression

	**Non-treated n = 14**	**Castration-resistant[a] n = 47**
**Age at diagnosis** (yrs)	74 (64-81)	69 (62-74)
**Age at metastasis surgery** (yrs)	74 (64-81)	72 (67-79)
**Serum PSA at diagnosis** (ng/ml)	580 (110-1900)	91 (42-560)
**Serum PSA at metastasis surgery** (ng/ml)	580 (110-1900)	340 (93-840)
**Gleason score at diagnosis**		
**6**	1 (7)	3 (6.4)
**7**	3 (21)	20 (43)
**8-10**	2 (14)	15 (32)
**Not available**	8 (57)	9 (19)
**Bicalutamide prior to surgery**		
Yes	-	28 (60)
No		19 (40)
**Chemotherapy prior to surgery[c]**		
Yes	-	8 (17)
No		39 (83)
**Radiation prior to surgery[d]**		
Yes	-	8 (17)
No		39 (83)
**Bisphosphonate prior to surgery[e]**		
Yes	-	6 (13)
No		41 (87)
**Ra-223 prior to surgery**		
Yes	-	5 (11)
No		42 (89)
**Follow up after metastasis surgery** (months)	31 (22-69)	5.7 (2.0-13)

Continuous values are given as median (25th-75th percentiles) and categorical values are given as numbers (precentages).

[a]Castration-resistant patients had disease progression after long-term androgen deprivation therapy including surgical ablation, LHRH/GNRH agonist therapy, and therapy with anti-androgens (bicalutamide).

[c]Chemotherapy included taxotere in 6 cases, estramustine in 1 case, and taxotere, carboplatin and etoposide in 1 case.

[d]Radiation towards operation site.
